# Restoring articular cartilage: insights from structure, composition and development

**DOI:** 10.1038/s41584-025-01236-7

**Published:** 2025-03-28

**Authors:** Alba Pueyo Moliner, Keita Ito, Frank Zaucke, Daniel J. Kelly, Mylène de Ruijter, Jos Malda

**Affiliations:** 1 Regenerative Medicine Center Utrecht, Utrecht, The Netherlands; 2 Department of Orthopedics, University Medical Center Utrecht, Utrecht, The Netherlands; 3 Department of Biomedical Engineering, Eindhoven University of Technology, Eindhoven, The Netherlands; 4 Department of Trauma Surgery and Orthopedics, Dr. Rolf M. Schwiete Research Unit for Osteoarthritis, University Hospital Frankfurt, Goethe University, Germany; 5 Trinity Centre for Biomedical Engineering, Trinity Biomedical Sciences Institute, Trinity College Dublin, Dublin, Ireland; 6 Department Clinical Sciences, Faculty of Veterinary Medicine, Utrecht University, Utrecht, The Netherlands

## Abstract

Articular cartilage can withstand substantial compressive and shear forces within the joint and also reduces friction during motion. The exceptional mechanical properties of articular cartilage stem from its highly organized extracellular matrix (ECM). The ECM is primarily composed of collagen type II and is pivotal in conferring mechanical durability to the tissue within its proteoglycan-rich matrix. Articular cartilage is prone to injury and degeneration, and current treatments often fail to restore the mechanical function of this tissue. A key challenge is replicating the intricate collagen–proteoglycan network, which is essential for the long-lasting restoration and mechanical durability of the tissue. Understanding articular cartilage development, which arises between late embryonic and early juvenile development, is vital for the creation of durable therapeutic strategies. The development of the articular ECM involves the biosynthesis, fibrillogenesis and self-assembly of the collagen type II network, which, along with proteoglycans and minor ECM components, shapes the architecture of adult articular cartilage. A deeper understanding of these processes could inform biomaterial-based therapies that aim to improve therapeutic outcomes. Emerging biofabrication technologies offer new opportunities to integrate developmental principles into the creation of durable articular cartilage implants. Bridging fundamental biology with innovative engineering offers novel approaches to generate more durable 3D implants for articular cartilage restoration.

## Introduction

Articular cartilage is a highly specialized connective tissue located in the synovial joint cavities lining the end of the epiphyses. The tissue consists of a thin hyaline cartilage layer (~0.1–2.5 mm, depending on location, age, sex and/or species^1–3^) which is important for the biomechanical function of the joint. The unique poroelastic properties of the tissue facilitate load transmission to the underlying bone and reduce friction to enable continuous smooth and pain-free movement^4^; however, the limited regenerative capacity of articular cartilage represents a major challenge for repair after injury or in disease. Therefore, understanding the composition and development of articular cartilage is crucial to improve therapeutic strategies.

The extracellular matrix (ECM) provides exceptional biomechanical support through its typical anisotropic [G] structure and composition^5^. Collagen type II is the main fibrillar protein in articular cartilage^6^, it forms heterotypic fibres [G] with collagen type XI (inside the fibre) and type IX (on the fibre surface) and interacts with numerous other proteins and small molecules^7,8^ In adult articular cartilage, these collagen fibrils run parallel to the articular joint in the superficial cartilage and curve perpendicular to the surface with depth, forming an arcade-like structure [G] (also known as “Benninghoff arcades”)^9^. Proteoglycans, predominantly aggrecan, are the second most abundant component in the articular ECM^10^. Aggrecan molecules are glycosylated with glycosaminoglycans (GAGs), mainly chondroitin sulphate and keratan sulphate (which are two repeating disaccharide polymers). Aggrecan molecules are negatively charged, which creates high osmotic potentials that draw water into the tissue, resulting in a gel-like composition with shock-absorbing properties^11^. The collagen fibre arcade-like structure limits aggrecan-induced swelling, which generates high swelling pressures that create the unique biomechanical property of cartilage that enables this tissue to withstand daily shear forces and impact loading^12^.

The regenerative capacity of articular cartilage is limited owing to it being avascular, aneural and dependent on nutrient diffusion from the synovial fluid^13^. Furthermore, although resident chondrocytes synthesise and maintain the ECM in their proximity^14^, the overall density of these cells throughout the cartilage tissue is low (~4.2×10^6^ cells per cm^3^)^15^. Thus, ECM degradation and articular cartilage injuries can cause pain, impaired mobility and a high risk of developing into osteoarthritis (OA)^16^.

Collagen structure forms during late foetal and early juvenile development, with minimal turnover in the adult tissue^17^. Upon articular cartilage injury, fibrotic scar tissue is deposited and mainly consists of mechanically inferior collagen type I^18^. Current treatments, including autograft implants^19^, cell-based (stem cell and chondrocyte transplantation)^20^ and biomaterial-based (often collagen type I scaffolds)^21^ therapies, fail to effectively restore the original ECM architecture in the long-term, highlighting the need for new approaches to restore tissue function^22^. Meanwhile, tissue engineering and 3D bioprinting technologies offer great promise for providing patients with living implants that can repair chondral and osteochondral defects^23,24^; however, although such 3D printed implants might show potential in the short-term, they do not restore the collagen architecture and the aggrecan gradient, meaning the implant will fail once the initial load-supporting materials have degraded^25^. The lack of long-term therapies highlights the need for a deeper understanding of the mechanisms that govern collagen alignment during embryonic and postnatal development. The cues that guide ECM composition and reorganization could provide valuable insights for novel biomimetic therapies that are capable of reproducing the long-term biomechanical function of the articular cartilage^25^.

In this Review, we explore the basic composition, structure and development of articular cartilage, with a particular focus on how the anisotropic composition and structure of the collagen and proteoglycan networks are shaped. We highlight potential strategies for directing collagen alignment in engineered tissues and aim to explore how novel biomimetic therapies could reproduce articular tissue anisotropy. Finally, we examine novel biofabrication technologies that hold promise for directing cartilage ECM alignment, with potential therapeutic applications for chondral and/or osteochondral implant engineering.

## The anisotropic nature of articular cartilage

Articular cartilage is a highly anisotropic tissue; at birth, the ECM is homogenous and predominantly isotropic [G] but during maturation the ECM composition and structure of non-calcified articular cartilage develops into different zones^9^. This anisotropy is essential for the tissue to withstand the complex mechanical forces in the joints.

### Collagen and proteoglycans in articular cartilage

**Figure 1:**
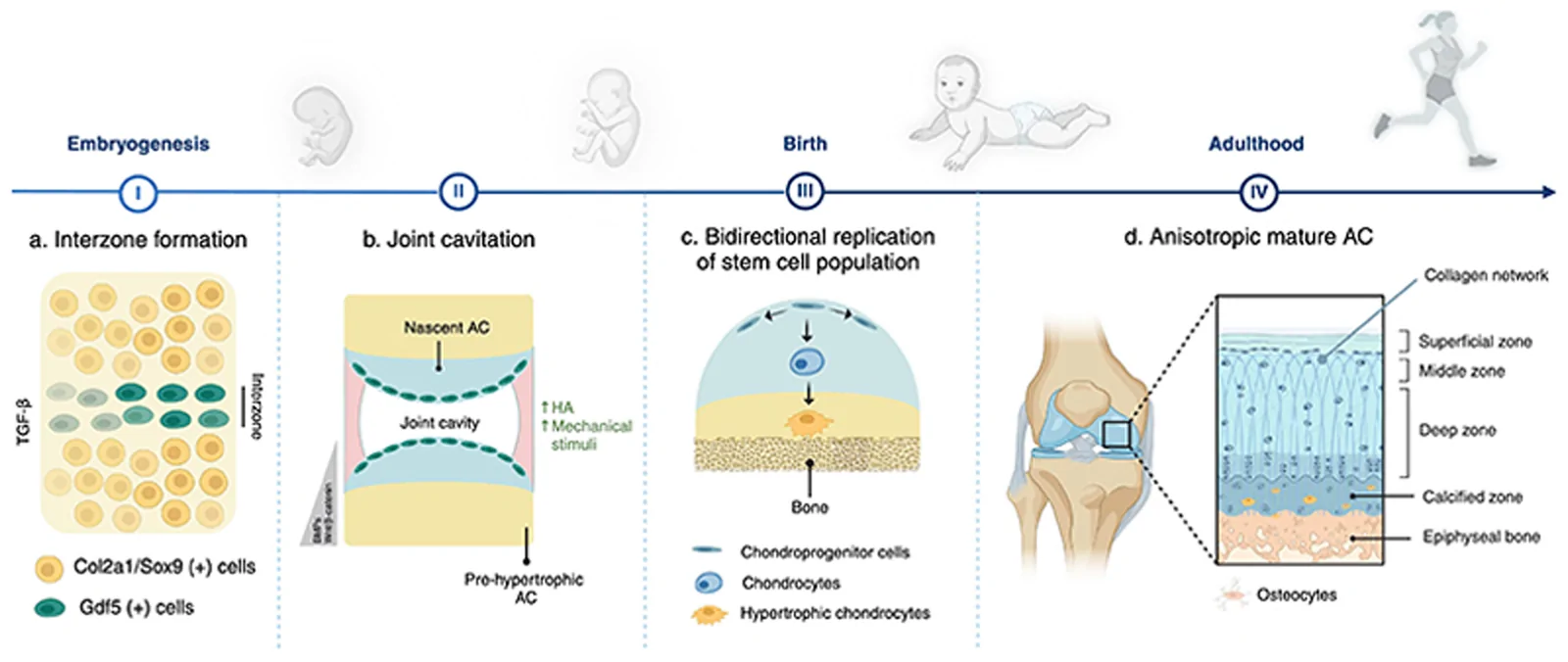
Development of knee articular cartilage from embryogenesis to adulthood. a, Interzone formation in the nascent limb occurs when flattened, condensed *Gdf5*^+61^ interzone cells emerge within pre-cartilage tissue that is composed of *Col2a1*^+^ *Sox9*^+^ cells^58,59^. b, Joint cavitation occurs when increased hyaluronan synthesis drives fluid influx, with mechanical stimuli and muscle contractions contributing to cavitation^62^. c, Chondroprogenitor cells at the surface of post-natal articular cartilage undergo bidirectional replication, producing a columnar progeny of chondrocytes and maintaining a population of self-renewing chondroprogenitors^26,56,67,93^. d, Upon maturation, the tissue exhibits zonal organization: the superficial zone has collagen fibres oriented parallel to the surface; the middle zone exhibits an isotropic fibre arrangement, and the deep zone contains vertically aligned collagen fibres, forming an arcade-like structure^9^. The innermost calcified zone, containing hypertrophic chondrocytes, anchors the cartilage to the subchondral bone, which hosts various bone cells, including osteocytes^66^.

Based on the orientation of collagen fibres, the zones of articular cartilage can be classified into the superficial zone (also known as the tangential zone), the middle zone (also known as the transitional zone) and the deep zone (also known as the radial zone (Figure 1) ^26^. The superficial zone is relatively thin^27^, with low proteoglycan content and fine collagen fibres aligned parallel to the surface, which provide resistance to both tensile and shear forces^28^. The collagen fibres parallel to the articular surface not only exhibit depth-dependent organization but also a distinct arrangement across the entire articulating surface (known as split-line orientation patterns [G])^29^. This organization aligns with the maximum tensile stresses experienced by the joint surface during motion and weight-bearing; high tensile strength occurs in a direction that aligns with these fibres^30^. Consequently, the articular cartilage surface also has anisotropic properties; for example, split-line orientation patterns are more consistent in areas that experience more contact than those that have less contact, which enhances the ability of the tissue to withstand and distribute mechanical loads across the joint surface^30^.

The superficial zone is rich in lubricin (also known as proteoglycan-4), a proteoglycan that contributes to articular surface lubrication (leading to a static and kinetic friction coefficient of 0.01 and 0.003, respectively^31^). Chondrocytes near the articular surface have a flattened morphology (Figure 1); articular cartilage progenitor cells (ACPCs) are also found in this area^32–34^. These multipotent chondroprogenitor cells make up 0.1-1% of the total cells in cartilage and are present in both young and adult cartilage^35–39^. In response to in vivo cartilage injury, ACPCs migrate to the damaged site, suggesting a potential role for these cells in tissue homeostasis and repair^40^. The high proliferative capacity of ACPCs and their ability to undergo chondrogenic differentiation^41–43^ make them promising candidates for therapeutic applications in neo-cartilage formation^39^.

In the middle zone, the orientation of collagen fibres is random, whereas in the deep zone – the thickest layer – fibres are aligned perpendicular to the surface (Figure 1). Compared with other zones, the deep zone contains the highest proteoglycan content and the thickest collagen fibres^55^, which anchor into the subchondral bone ^44^. Oval chondrocytes are distributed throughout this zone and are arranged in vertical columns^33^ (Figure 1). The deepest layer is the calcified cartilage, which anchors to the underlying epiphyseal bone. Here, chondrocytes are sparse and hypertrophic^45^, and collagen type X is abundant^46^. This layered organization has remained preserved across the evolution of terrestrial mammals^27,47,48^.

The ECM is divided into three regions based on chondrocyte proximity: the pericellular matrix (PCM, 2-4 µm), the territorial matrix and the interterritorial matrix. The PCM is in direct contact with chondrocytes and differs from the territorial and interterritorial areas in structure, function and composition. The PCM controls chondrocyte phenotypes, acts as a growth factor reservoir and is important for mechanotransduction^49^. The major components of the PCM are perlecan^50^, collagen type VI^51^ and fibronectin^52^. Collagen type VI forms a beaded microfibrillar network [G] that anchors the chondrocytes to the surrounding ECM and mediates cell–matrix interactions via integrin cell receptors^53^. In the PCM, collagen fibres are oriented in a basket-like pattern around the chondrocytes^54^. The combination of the PCM and chondrocytes is referred to as the chondron^49,55^. The chondron is immediately surrounded by the territorial matrix, which is rich in proteoglycans, and the interterritorial matrix, which comprises the majority of the ECM.

### The bottom-up development of articular cartilage

During embryonic development, articular cartilage formation begins with the establishment of the synovial joints^56^. Lineage tracing studies in mice have provided insights into this complex process. Joint establishment starts with the formation of the interzone, a region that initially consists of Col2a1^+^ Sox9^+^ cells that are recruited from the mesenchymal condensation of the developing limb bud^57^. These mesenchymal progenitors flatten and compact to settle in the future joint^58^ (Figure 1a). As development continues, the interzone is populated by cells that express *Gdf5*, including cells that later migrate into the developing limb^59^. *Gdf5*^+^ cells are responsible for forming articular cartilage and other joint structures (such as the ligament and the meniscus). Several hypotheses have been proposed to explain how *Gdf5* expressing cells give rise to tissue divergence^60^. This process is followed by joint cavitation [G] ^61^. Joint cavities form from fluid influx that is induced by the upregulation of hyaluronan synthesis^62^, which causes swelling pressure that separates the interzone into two opposing tissues (Figure 1b). Mechanical stimuli, via foetal muscle contraction, also has a role in joint cavity formation^63^. Interzone and joint cavitation are regulated by numerous factors and signalling pathways^58^. After the synovial joint space is formed, the articular ends start a morphogenesis process, in which early cartilage shape, organization and structure are established^64^.

Chondroprogenitor cells that are present at the surface of the nascent [G] joint contribute to the development of all the layers of articular cartilage and supply rapidly proliferating daughter cells that move down vertically to build the ECM (Figure 1c). These chondroprogenitor cells express *Prg4* and are hypothesized to be descendants of interzone cells that downregulate *Gdf5* expression after birth^65^. The descendant chondrocytes will migrate further and undergo hypertrophy [G] and apoptosis after building an ECM that is invaded by blood vessels and various bone cells, creating calcified bone tissue (known as the secondary ossification centre). The hypertrophic chondrocytes that do not die can transition into osteogenic cells^66^.

Some daughter cells will halt hypertrophy as they migrate downwards and retain a chondrocyte phenotype, forming a layer of non-ossified cartilage ^67^. The tidemark [G] is the boundary between the non-calcified and calcified zones (Figure 1d). Thereafter, a bottom-up growth of articular cartilage tissue occurs above the calcified layer, which is gradually built up by chondrocyte proliferation and ECM deposition^58,68,58,69^. During late embryonic and postnatal tissue growth, articular cartilage undergoes further morphogenesis, marked by the realignment of the ECM. Collagen fibres are initially more isotropic [G] but eventually become perpendicular to the surface in the deep zone and parallel to the surface in the superficial zone (Figure 1d).

## Collagen type II fibres in the articular cartilage matrix

Collagen is present in the articular ECM in the form of heterotypic fibres; typically, collagen type II is packed together with collagen type XI creating tightly packed, resilient networks^6^. This process is facilitated by the presence of other proteins that interact with these fibres, such as collagen type IX (on the fibre surface)^68^ and small leucine-rich proteoglycans^69^, which not only facilitate assembly, but also regulate fibre diameter. For simplification, hereafter we refer to heterotypic cartilage collagen fibres as collagen type II fibres. Collagen biosynthesis and fibrillogenesis [G] contribute to the growth and integrity of the tissue^70^. Therefore, to elucidate how this complex architecture is achieved in the mature tissue, it is important to understand the key elements of collagen synthesis, its release into the ECM and the process of fibrillogenesis into the existing network.

### Collagen biosynthesis

**Figure 2:**
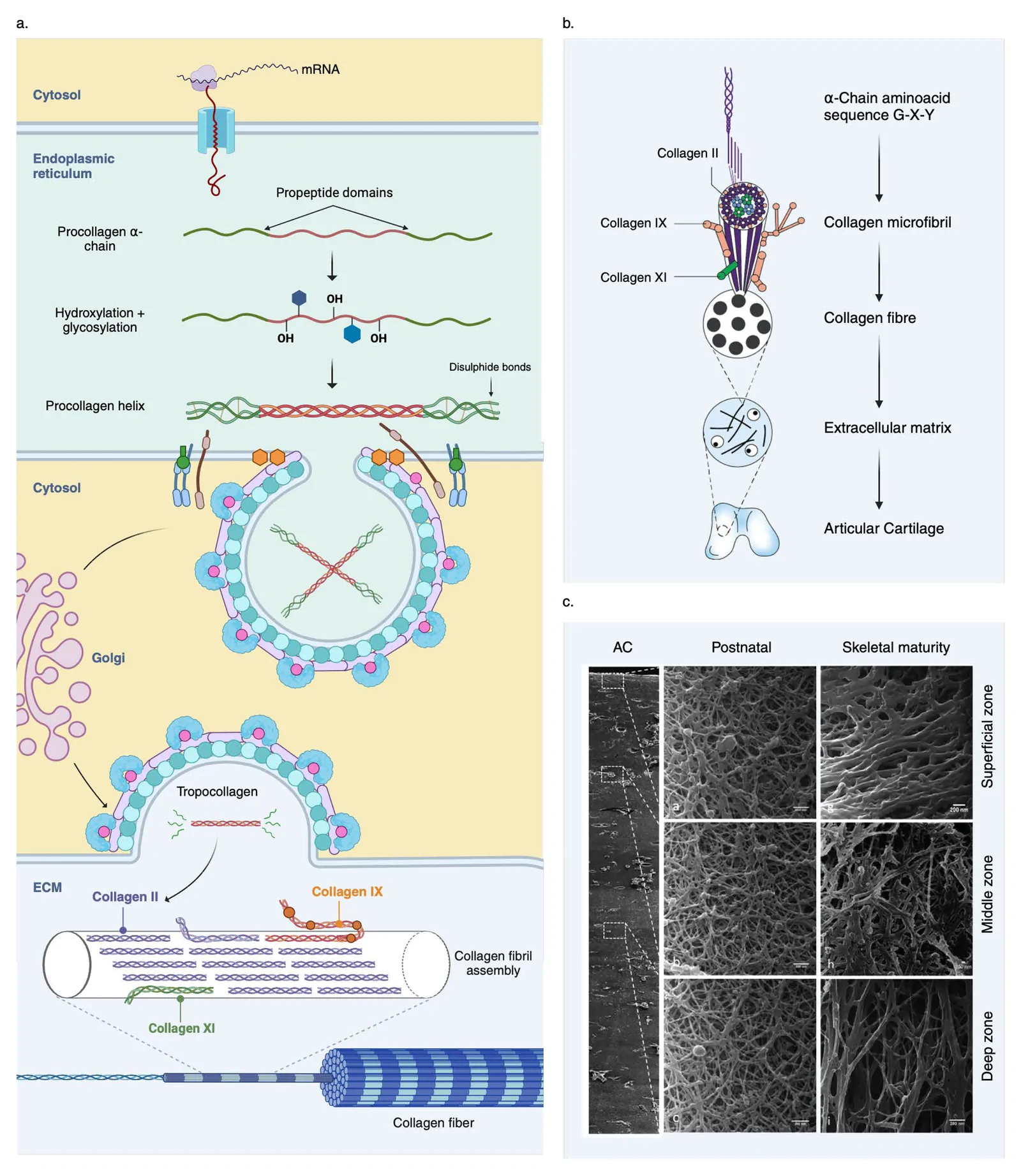
Collagen fibrillogenesis a, Procollagen is released into the endoplasmic reticulum (ER). Procollagen α-chains have tripeptide glycine-X-Y (G-X-Y) repeats that are common to all collagens and are essential for the assembly of the α-chains into a procollagen triple helix (the X and Y residues are typically proline and hydroxyproline respectively^74^). Prior to forming the procollagen triple helix, these chains undergo post-translational modifications in the lumen of the ER^251^ (hydroxylation and glycosylation). The procollagen helix is then transported to the Golgi where it is processed further and finally secreted into the extracellular matrix (ECM)^73^. Propeptides (green) are cleaved to form tropocollagen molecules, that then self-assemble into fibrils, incorporating as collagen type XI (red) and fibril-associated collagens (such as collagen type IX (yellow))^76^. b, The collagen fibril core is formed by two collagen type II and two collagen type XI microfibrils, which are surrounded by 10 additional collagen type II microfibrils. Multiple collagen fibrils organize into larger structures, known as collagen fibres. c, During cartilage maturation, the number of crosslinks within collagen fibres increases, leading to the formation of thicker and more mechanically robust fibres^88^. Collagen fibres also undergo reorganization, in the superficial zone, fibres align parallel to the surface, whereas in the deep zone, they adopt a vertical orientation^9^. mRNA, messenger RNA; OH, hydroxyl group.

Collagen type II biosynthesis begins with the transcription of the *COL2A1* gene into two mRNA isoforms (IIA and IIB) by alternative splicing. Chondroprogenitor cells synthesize collagen IIA and mature differentiated chondrocytes synthesize collagen IIB^71^. Collagen IIA contains an additional exon, leading to a cysteine-rich domain in the N-pro-peptide region, which interacts with TGF-β and BMPs. Collagen IIA is expressed during the early stages of cartilage development^72^. Collagen IIB is synthesized by adult chondrocytes and is the major form of collagen in adult articular cartilage^71^. Subsequent translation of the IIB mRNA isoform results in the formation of the procollagen [G] α-chains (Figure 2a). Three procollagen α-chains coil around each other to generate a procollagen right-handed triple helix (Figure 2a). Centrally located glycine residues allow tight packing of the helix^73^. The N-terminal and C-terminal are known as pro-peptides and do not assemble into the triple helix but are crucial for extracellular polypeptide secretion and helix formation, as disulphide bonds form between these regions^74^. Procollagen is then released into the ECM and the pro-peptides are cleaved by proteinases before being deposited together with collagen XI into the collagen fibrils^74^ (Figure 2a). Pro-peptide removal leads to reduced solubility of the procollagen, which then assembles into a highly organized tropocollagen [G] structure. Finally, adjacent tropocollagen molecules assemble in a highly intricate pattern to build fibrils and fibres that have a large diameter (Figure 2a)^74^.

### Fibrillogenesis and self-assembly

Fibrillogenesis is a step-by-step process that begins when chondrocytes release collagen molecules. These individual collagen molecules (~300 nm long and 1.5 nm in diameter)^75^ fuse together laterally or linearly, forming large fibrils with the collagen triple helices aligned in the same direction within the fibril^76^. Different populations of thin (~16-20 nm in diameter) and thick fibrils (~40 nm in diameter) are described in the articular ECM^76^. Collagen type XI is predominantly found in the thinner fibrils, acting as a fibrillogenesis nucleator [G]. Two collagen type II and two collagen type XI microfibrils form the fibril core, surrounded by ten collagen type II microfibrils (known as the ‘10+4’ arrangement)^7^ (Figure 2b). Initially, smaller fibrils are formed, which interconnect over time to form larger fibril bundles (also referred to as fibres, composed of multiple fibrils). Collagen type XI can also crosslink with other collagen type XI molecules^77^. Other types of collagens, such as type IX, are present in smaller amounts and help to stabilize the heterotypic collagen II–XI fibrils^74^. Collagen type IX is a fibril-associated collagen with interrupted triple helices. This collagen forms covalent bonds with collagen type II and other type IX molecules and also crosslinks collagen type II with other ECM components^78,79^. Collagen type IX is located on the surface of thin fibrils in adult cartilage, disappearing from the thicker fibres as cartilage matures ^8^. Its N-terminal non-collagenous domain 4 projects away from the collagen fibrils^80^, interacting with ECM components, such as biglycan^78^ or fibronectin^79^. Collagen type II, IX and XI ratios can vary, with an 8:1:1 ratio present in thin fibrils during development^81^

Collagen fibre diameter increases with growth and cartilage depth^82^ (Figure 2c). Fibril synthesis is influenced by various mechanisms, including growth inhibition, degradation and interactions with small proteoglycans that regulate fibril formation. Additionally, limited fibril diffusion in proteoglycan-rich areas can further slow fibril growth^83^. Thicker fibres, which lack collagen IX and XI, often contain decorin, a small proteoglycan that binds non-covalently and maintains proper separation between fibres — especially in deep and superficial regions where fibres run parallel to each other^8^. Decorin also enhances adhesion between aggrecan molecules and between aggrecan and collagen^8,84^. Therefore, collagen fibrillogenesis takes place on a densely populated matrix, where small proteoglycans and other ECM components create a natural molecular crowding [G] effect, that catalyses fibrillogenesis^85^. This molecular crowding effect, and the high affinity of collagen II for molecules that regulate fibrillogenesis, contribute to fibril formation^86^.

Cartilage collagen is further interconnected by crosslinking, which is mostly facilitated by the enzyme lysyl-oxidase (LOX)^87^. As cartilage matures, both the nature and number of crosslinks increase, contributing to the increased stiffness and brittleness that is associated with aging^88^. Initially, the collagen fibrils contain reducible crosslinks [G] (specifically, divalent ketoimines^89^), which gradually convert into non-reducible crosslinks [G] (specifically, trivalent pyridinolines). Besides the regulation of fibre formation and crosslinking, fiber degradation is also essential for shaping the collagen network. Chondrocytes produce matrix metalloproteinases (MMPs) that specifically target and degrade collagen, destabilizing fibrils by targeting regions involved in crosslinking. Additionally, other mechanisms, such as the oxidative state of the ECM or advanced glycation end-products, influence the balance of crosslinking in mature collagen fibres^88^. Since mature articular cartilage collagen is slowly renewed and primarily deposited during growth^17^, its crosslinking is crucial for joint longevity and resistance to mechanical failure. The identification of the factors that drive initial crosslink formation and those that regulate crosslinking could elucidate why joints fail earlier in some individuals than in others. Altogether, larger-diameter fibres, collagen structure and collagen–aggrecan and collagen–collagen crosslinking further enhance ECM stiffness, leading to increased resistance to compression with maturation, especially in the deeper zones. These insights could inform future tissue engineering approaches.

## Postnatal collagen structure reorganization

Considering the composition, structure, postnatal development and poroelastic behaviour of articular cartilage, it is evident that collagen structure and architecture have a fundamental role in shaping tissue mechanics. Collagen production, fibrillogenesis and tissue growth are crucial for this architecture; however, the exact mechanisms underlying the formation of the collagen arcade-like structure remain elusive. Current research efforts face two major challenges^90^, firstly, it is difficult to directly visualize collagen network formation and, secondly, the pace of collagen synthesis is relatively slow. Several hypotheses have emerged to explain how the structure of articular collagen develops. Data from studies on collagen reorganization in vertebrates (such as sheep^91^, pigs^82^, horses^92^, rabbits^93^ and opossums^94,95^) suggest that there are similarities in the remodelling processes among different joints and different terrestrial species. However, the articular cartilage collagen structure found in aquatic mammals is strikingly different, with a more random orientation, probably owing to the loss of terrestrial loading-based evolutionary pressure^48^. Therefore, articular cartilage development enables the tissue to adapt to increased loading^96^ through the reorganization of the collagen type II fibres in the late prenatal and early juvenile stages up until skeletal maturity^93^. Notably, collagen molecules incorporated into fibres during development persist throughout life^17^. Termination of growth activity in the epiphyseal bone usually coincides with the maturation of the articular cartilage structure and the development of an anisotropic collagen organization^93^. This section describes the possible mechanisms that lead to collagen reorganization and highlights the need for further research to fully elucidate the orchestration of this complex process.

### Collagen network formation in the deep zone

**Figure 3:**
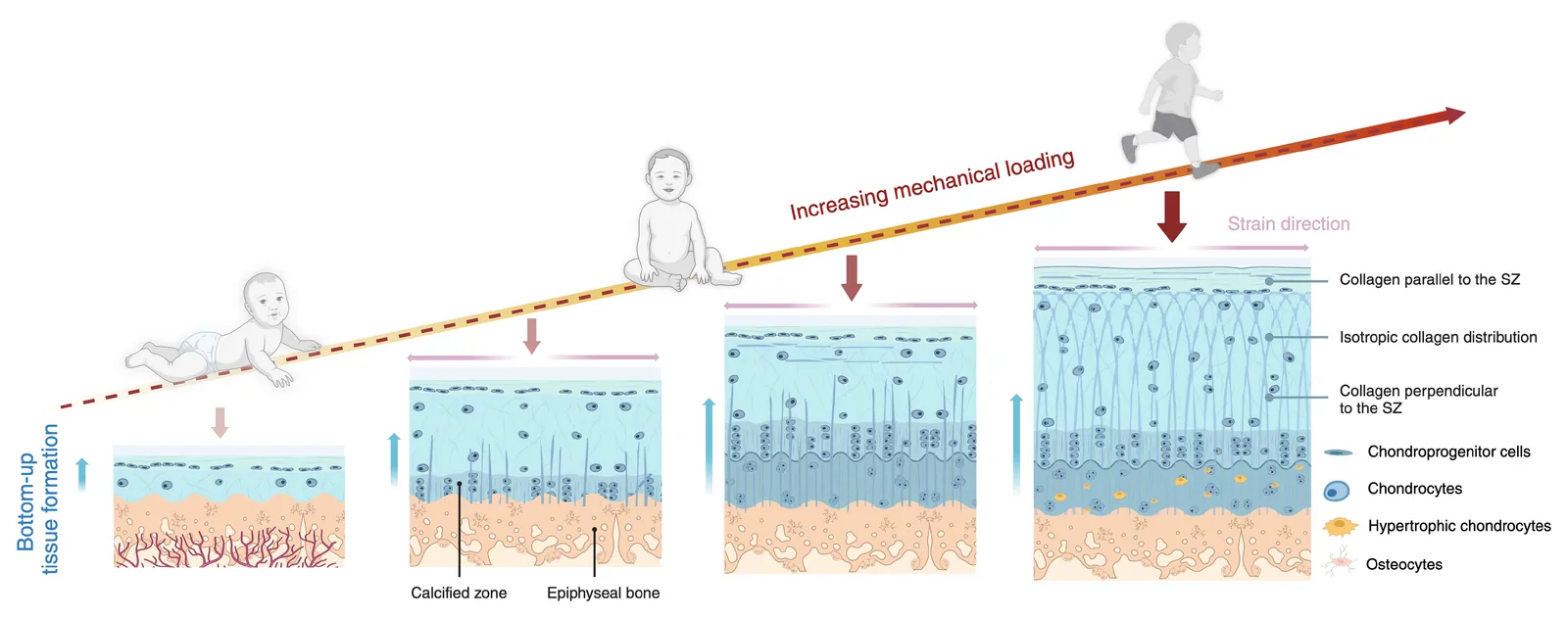
Proposed mechanism for the guidance of collagen type II fibres in articular cartilage During postnatal development, bottom-up tissue growth is not driven by internal tissue remodeling, but rather by tissue neoformation due to the columnar division of chondrocytes in the proliferative zone, leading to a bottom-up tissue growth that pulls collagen fibres into a perpendicular alignment relative to the cartilage surface in the deep zone^101,102^. Additionally, increasing mechanical load promote the alignment of collagen fibres parallel to the surface in the superficial zone^116,120–123^. The articular surface also grows and stretches during development, creating tangential strains that pull the collagen fibres parallel to the surface^101^. At the same time, articular cartilage acts as a surface growth plate for the underlying epiphyseal bone, as chondrocytes undergo hypertrophy creating calcified bone tissue.

For fibrous tissues, such as tendons, fibroblasts align with the principal stress direction, producing collagen type I fibres that extend into the matrix in the same orientation. This alignment occurs within specialized extra-cellular cell membrane invaginations, known as fibripositors, in which fibres self-assemble after collagen monomers are secreted by fibroblasts^97^. Unlike collagen type I, active guidance of collagen type II by chondrocytes has not been demonstrated. In fact, the orientation of collagen type II fibres is considered a cell-independent process^98^. Thus, given that the chondrocytes in articular cartilage do not polarize, are encapsulated in a PCM and are sparsely distributed within the articular ECM, these cells are unable to directly reorganize collagen fibres or guide their orientation. Nevertheless, collagen alignment is probably indirectly related to tissue growth during postnatal development. Notably, collagen type II fibres are anchored to the subchondral bone^99^. During postnatal development, chondrocyte progenitors give rise to daughter cells that synthesize new ECM, which drives articular cartilage growth from the bottom-up^100^. During this process, collagen fibres that are anchored to the subchondral bone could be pulled upwards with perpendicular-to-surface tissue growth, and new collagen monomers integrated into the fibres in this direction^101^. This mechanism has been demonstrated in vitro by stimulating neo-cartilage bottom-up tissue growth in a transwell system^101,102^. Therefore, postnatal cartilage growth could be responsible for the arrangement of fibres in the deep zone, not directly by cell deposition, but by indirect effects of tissue growth (Figure 3), which would explain the perpendicular orientation of fibres from the subchondral bone towards the surface in the deeper zones of the articular cartilage^101^.

### Collagen network formation in the superficial zone

Tightly packed, tangentially oriented collagen fibres provide crucial support for shear-resistance and normal mechanical function of the articular cartilage^103^. In some species (mostly those that require locomotion immediately after birth^104^), the mechanical forces that occur from initial movements are an essential factor in joint formation^105–107^, and lack of intrauterine foetal or postnatal joint motion (which can be caused by conditions such as developmental dysplasia of the hip or congenital hip dislocation^108^) results in abnormally shaped^109–111^ or underdeveloped joints^108,112^. Several theoretical models that predict the structure of collagen networks across different tissues are built on the principle that collagen orients in the direction that maximizes its function^113^ (such as resistance to the positive principal strain [G] ^114,115^). In articular cartilage, this principle is in line with in vivo^116,117^ and *in* vitro^118^ studies that suggest that increased loading during late prenatal and postnatal development could be responsible for the parallel collagen fibre alignment on the cartilage surface (Figure 3). Research on the postnatal development of calf femoral head cartilage demonstrated the presence of split lines predominantly in regions subject to major load bearing stress^117^. These split line patterns, which indicate the predominant tangential collagen orientation, only appear during postnatal development within the superficial zone, and are absent in newborn cartilage, suggesting a progression linked to mechanical stimuli^119^. Another study highlighted that porcine knee cartilage from meniscus-covered regions, which experiences lower mechanical loads, has less parallel collagen alignment in the superficial zone, with more randomly oriented fibres and a lower density, compared with regions that are not covered by the menisci^116^.

Overall, newly synthesised collagen fibrils are dispersed in the surrounding ECM. Upon collagen release, the loose fibrils are assembled in an isotropic manner into the existent network^70^. Therefore, when the superficial area experiences increased loading after birth, the collagen fibres begin to arrange themselves in a parallel pattern, probably owing to external loading forces^90,101^. This hypothesis is consistent with research that shows that the surface collagen fibre reorganization coincides with increased postnatal joint loading^116,120–123^. In addition, as the ECM in the deep zone expands, the superficial zone becomes compressed, which results from the combined effect of joint loading and bottom-up growth of deeper tissues^101^. Mechanical loading might also facilitate this compression by causing the release of water in the surface zone rather than in the deeper zones, in which the proteoglycan content and therefore osmotic potential are higher^28,124^. Also, the articular surface grows and stretches during development, creating tangential strains that could potentially pull the collagen fibres parallel to its surface^101^ (Figure 3).

Taken together, these observations point towards a biomechanically driven hypothesis of collagen realignment, in which the orientation of collagen fibres is influenced by the forces generated within the ECM in response to tissue growth (which guides collagen alignment in the deep zone) and physiological loads (which guides collagen alignment in the superficial zone)^101^. Further understanding of this mechanically driven process could help to recreate collagen structures and achieve durable functional regeneration of the articular cartilage.

## Proteoglycans in the articular cartilage matrix

Alongside fibrillogenesis, self-assembly and postnatal reorganization of collagen type II, the collagen network interacts with various other ECM components, which determines the overall mechanical properties of articular cartilage. The interplay between proteoglycans and collagen type II gives articular cartilage its unique poroelastic behaviour^125^. The water-attracting properties of the GAGs are constrained by the arcade-like collagen fibre network, building up a swelling pressure and enabling the tissue to withstand repeated mechanical loads over a lifetime. In the absence of external forces, collagen tension balances the swelling pressure created by negatively charged GAGs. This internal tension places the collagen network under stress, even when unloaded. Under compression, the pre-stressed collagen fibres, especially in the deep zone, in which fibres are thicker, highly ordered, and aligned with the load direction, provide resilience, unlike the more disorganized fibres in immature cartilage^126^. As proteoglycan density decreases towards the superficial zone, the collagen network experiences less swelling pressure, leading to lower fibre pre-strain in the middle zone ^127,128^. Under physiological articular loading rates, the pressurization of the fluid retained by the GAGs primarily supports the load^10^. In addition to generating swelling pressure, the collagen network restricts proteoglycan movement, thereby resisting the outflow of interstitial fluid from the articular cartilage (which has low permeability)^90^. Aggrecan is the most abundant proteoglycan in articular cartilage and has a crucial role in load bearing. Its interaction with the collagen type II network is mediated through various molecular forces, including Van der Waals, hydrophobic and hydrogen bonds and electrostatic interactions between the GAGs and positively charged amino acids on the collagen fibres^129^. Although aggrecan is the dominant proteoglycan found in articular cartilage, a range of smaller proteoglycans and glycoproteins also have an essential role in regulating collagen fibre organization and tissue homeostasis^68,130^. These small proteoglycans, such as biglycan, also contribute to more complex bridging interactions between aggrecan molecules and collagen fibres^84^.

### Aggrecan biosynthesis and deposition

**Figure 4:**
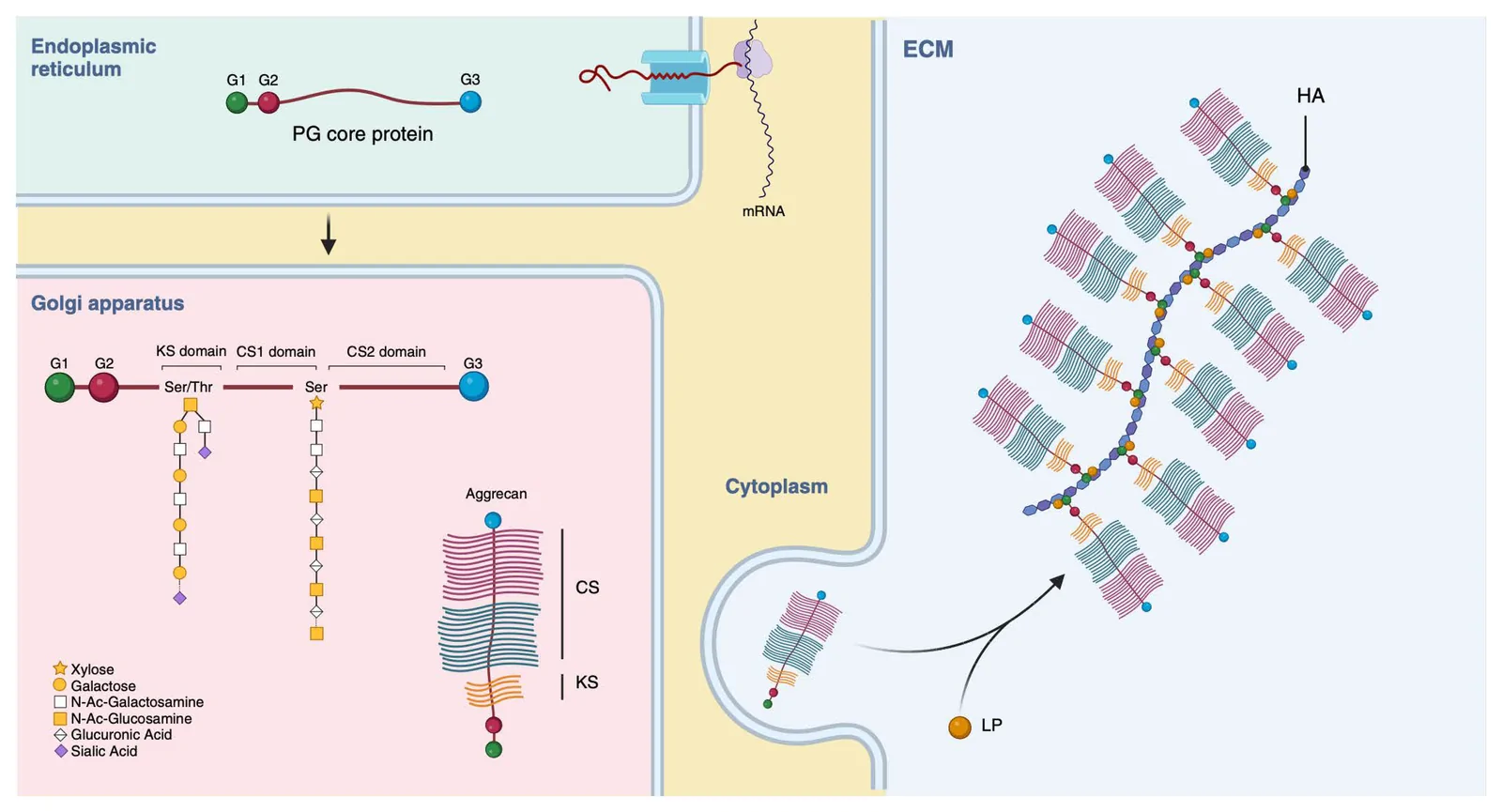
Proteoglycan aggrecan synthesis. The aggrecan core protein contains three globular domains (G1, G2, and G3) and a non-globular region between G2 and G3 for GAG attachment. The aggrecan core protein is released into the endoplasmic reticulum (ER). Glycosylation occurs in the Golgi, where specific sugars are sequentially added to different non-globular domains to form keratan sulphate (KS) and chondroitin sulphate (CS) negatively charged GAGs^135^. Following glycosylation, aggrecan is secreted in vesicles into the extracellular matrix (ECM). In the ECM, aggrecan binds to hyaluronan through the link protein, forming bottle-brush-shaped proteoglycan aggregates^136^. mRNA, messenger RNA; Ser/Thr, Serine/Threonine.

Aggrecan is composed of a core protein that is covalently bound to sulphated GAGs, mainly chondroitin sulphate and keratan sulphate. The core protein of the aggrecan monomer is transcribed from the *ACAN* gene. The aggrecan core protein has five domains^131,132^: three globular domains (G1 and G2 at the N-terminal, and G3 at the C-terminal), an interglobular domain and an extended central domain with serine-glycine repeats (Figure 4), in which the chondroitin and keratan sulphate chains bind^133^. Glycosylation occurs in the Golgi, where the first sugar of the GAG chain is added^134^. Various glycosyltransferases elongate the keratan sulphate and chondroitin sulphate chains (Figure 4)^135^. In humans, each aggrecan molecule contains 30-60 keratan sulphate chains and up to 100 chondroitin sulphate chains. After synthesis, proteoglycans are secreted into the extracellular space via secretory vesicles. Aggrecan is found in aggregates in the articular ECM, hence the name of this proteoglycan, it binds non-covalently around a central hyaluronan filament through the N-terminal G1 domain. This interaction between aggrecan and hyaluronan is stabilized by the presence of the link protein^136^ (Figure 4). Hyaluronan is formed in the cytosol and directly released to the extracellular space, forming a layer around chondrocytes^137^.

### Aggrecan structure and distribution

As previously mentioned, collagen fibres in the articular cartilage exhibit an anisotropic structure, and aggrecan has a depth-dependent gradient. At the surface, proteoglycan content is relatively low, but it increases with depth^5^, which raises swelling pressure and water content in the middle and deep layers. In the calcified cartilage, proteoglycan content decreases again, although aggregates are larger and more saturated with aggrecan^137^. These gradients influence tissue permeability, as negatively charged GAGs retain water which impedes fluid flow; the availability of soluble growth factors and cytokines are also varies with depth. The differential distribution of aggrecan is partially driven by the metabolic profile of chondrocytes and their depth-specific proteoglycan synthesis; the response of chondrocytes to mechanical loading varies across tissue zones^138^. Surface chondrocytes experience mostly shear and tensile forces, whereas deep zone chondrocytes endure compression. In vitro evidence suggests that such distinct loading regimes promote specialized chondrocyte behaviour^139^. Moreover, aggrecan from the postnatal matrix has fewer fragmented monomers compared with adult cartilage, even in healthy adults, suggesting that this characteristic might be part of a natural homeostatic process^140^ (Supplementary Figure 1). Aggrecan fragmentation, even if physiological, becomes pronounced during OA, indicating its role in disease progression^141^. In addition to the fragmentation of the core protein, the mean size of the GAG chains also decreases with aging and in OA^142^.

### Other proteoglycans and glycoproteins in cartilage

**Figure 5:**
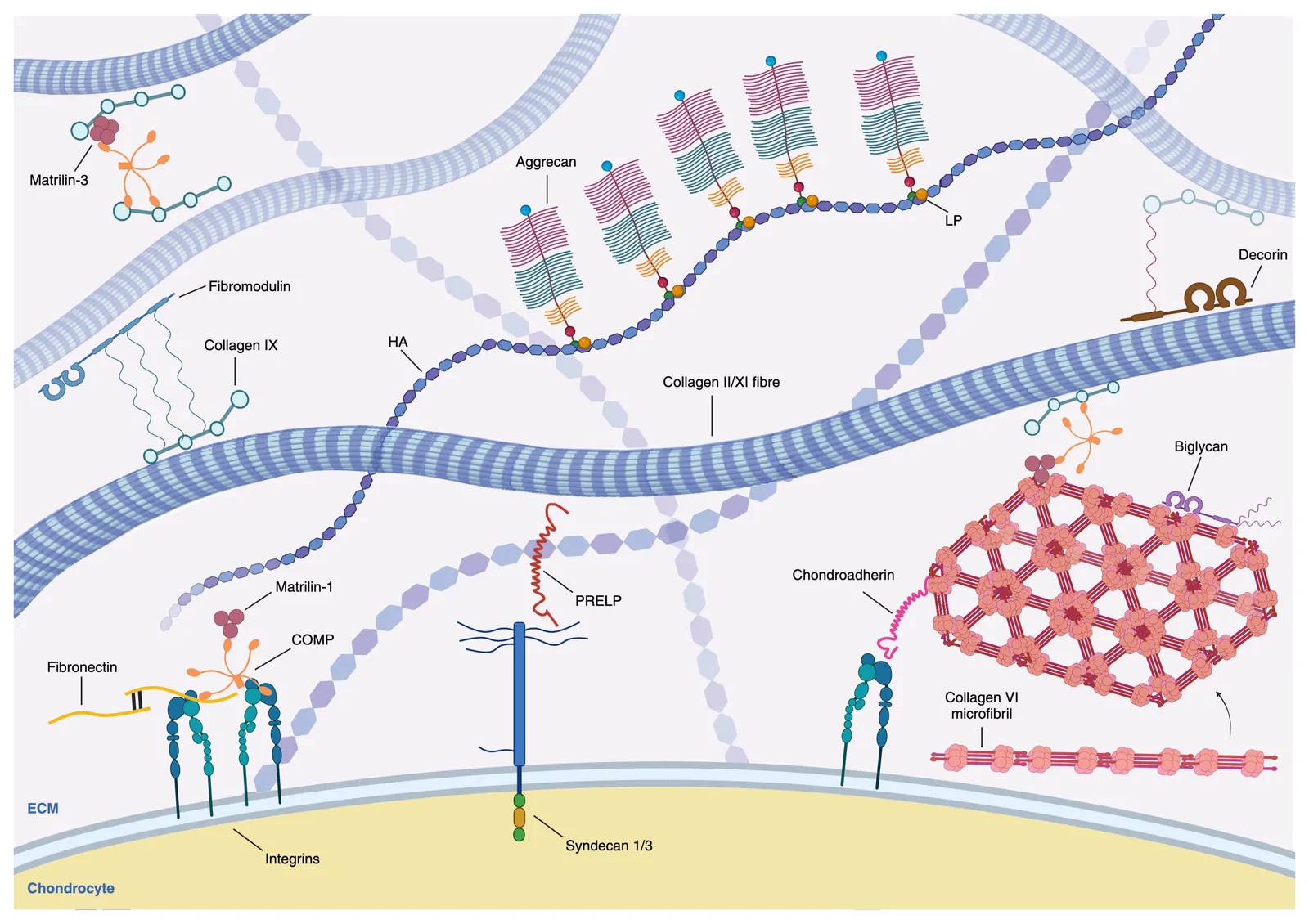
The main components of articular cartilage ECM The extracellular matrix (ECM) of articular cartilage contains numerous components (only the main components are shown here, see Table 1 for a comprehensive list of ECM components). Heterotypic collagen fibres (blue) form a fibrillar collagen network. Aggrecan molecules bind to hyaluronan via the link protein, assembling into large bottle-brush aggregates that create a hydrated poroelastic matrix. Various interactions between collagen fibres and other minor collagens (such as collagen VI and collagen IX), proteoglycans (such as decorin, biglycan, fibromodulin, chondroadherin and PRELP), glycoproteins (such as martilin-1, matrilin-3, fibronectin and COMP) and chondrocytes (via cell-surface receptors, such as integrins and syndecans), ensure cohesion and structural integrity of the tissue. COMP, cartilage oligomeric matrix protein; PRELP, proline/arginine-rich end leucine-rich repeat protein.

The interaction between collagen type II and large proteoglycans, such as aggrecan, has a crucial role in tissue stiffness and resistance to deformation. This interaction is, in many cases, indirect and mediated through protein complexes consisting of small proteoglycans and glycoproteins. Small proteoglycans such as versican, biglycan and fibromodulin, are essential for maintaining tissue integrity^143^. They closely interact with the arcade-like collagen network and perifibrillar adapter proteins, such as matrilins, regulating collagen fibre diameter and ensuring cohesion within the ECM^83,144^ (Figure 5). As summarized in Table 1, these proteoglycans, glycoproteins and small proteins are essential for collagen fibrillogenesis, mechanical stabilization and ECM crosslinking.

## Attempts to restore cartilage mechanical function

**Figure 6:**
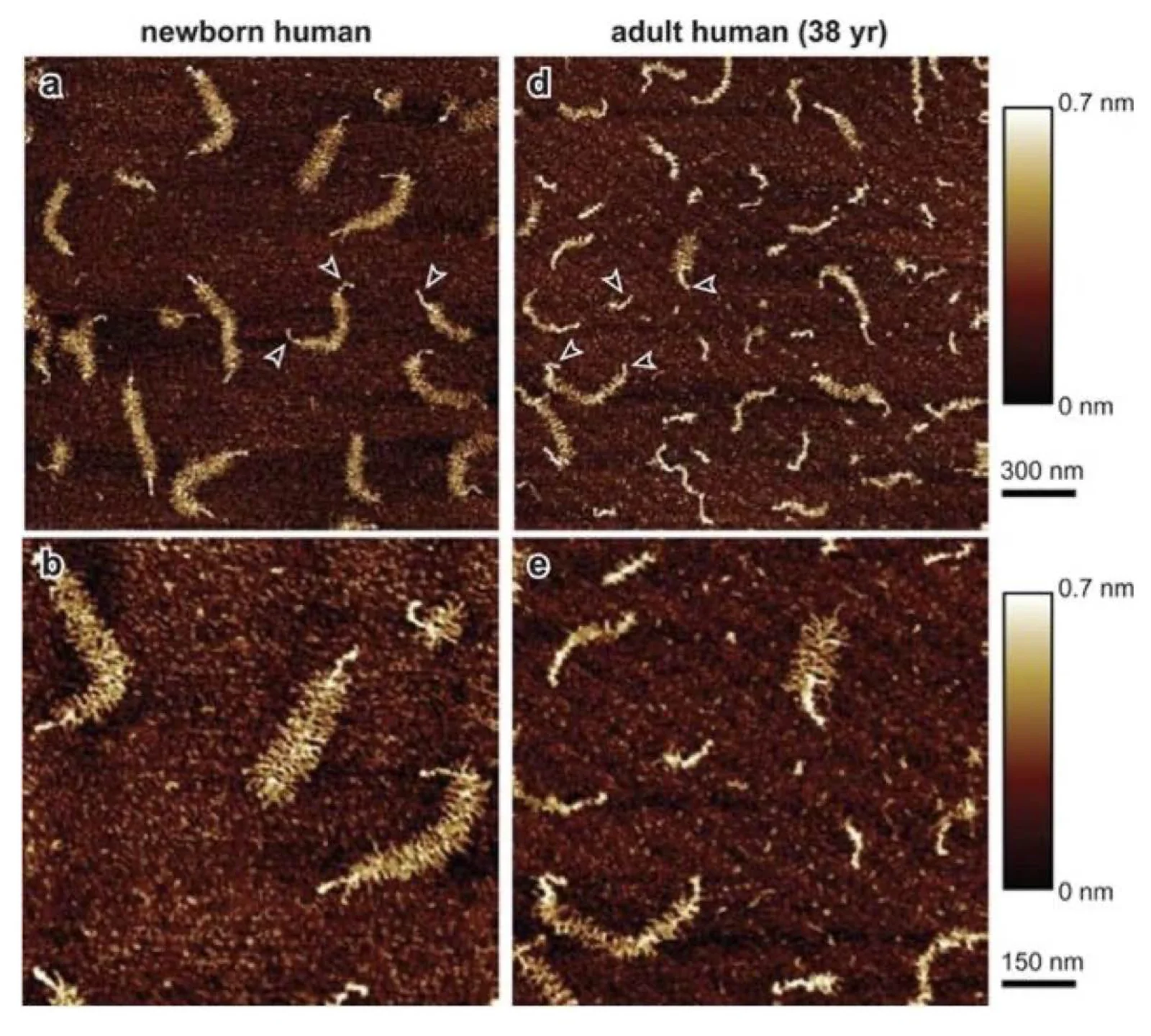
Strategies for guiding collagen in articular cartilage restoration and biofabrication a, Traditional approaches for articular cartilage restoration include autograft^19^ or allograft^146^ transplantation, autologous chondrocyte injection^20^ and the incorporation of chondrocytes into hydrogel-based scaffolds to enhance chondrogenesis and ECM production^151^. These hydrogels can be extrusion-printed to create depth-dependent gradients^163^. b, Fiber reinforcement technologies, such as melt electrowriting (MEW), enhance the mechanical properties of hydrogels, bringing the implants closer to mimicking native tissue mechanics^169^. Moreover, spatial confinement of the fused spherical aggregates can give rise to an arched orientation of collagen in some regions^171^. d, Novel technologies for collagen alignment aim to develop implants with arched collagen networks and aligned collagen fibres, enhancing long-term mechanical properties. One approach involves mimicking tissue growth to achieve vertical alignment of collagen type II in the deep zone by guiding ECM production through spatially directed TGF-β stimulation^101^. Other approaches focus on fabricating anisotropic hydrogels using novel technologies (e.g., filamented light biofabrication^212^ or magnetic microgel alignment^214^), creating aligned scaffolds that can guide collagen deposition in in pre-cell-seeded implants. These approaches aim to produce implants with aligned collagen fibres and enhanced long-term mechanical properties.

Once skeletal maturity occurs, the collagen matrix cannot be further replaced^145^. This lack of remodelling means that any damage to the arcade-like structure of collagen is essentially permanent^25^. Besides the limited regenerative capacity of articular cartilage, the absence of collagen matrix replacement and the lack of a collagen architecture accounts for the consistent failure to durably regenerate articular cartilage. Traditional approaches, such as autograft^19^ and allograft^146^ transplantation, have been used as a biological substitute for the damaged osteochondral tissue (Figure 6a). Although these approaches include a replacement for the arcade-like network, they do come with considerable restraints. Besides potential limitations in availability, autologous transplantation can lead to tissue morbidity and dysfunction at the donor site^147^, whereas allogeneic transplantation can trigger an immune response^148^. Moreover, structural integration of the grafts into the host tissue^149^ can be a challenge and treatments will often fail in the long-term. In view of the aging population, there is therefore an urgent need for more durable approaches for articular cartilage restoration.

Novel clinical approaches, such as cell-based therapies, which involve the use of stem cells or chondrocytes (including expanded chondrocytes)^150^, are combined with soft hydrogel-based scaffolds to keep the cells in place and provide infill for irregularly shaped defects to promote neocartilage tissue formation^151^ (Figure 6). Hydrogel materials, whether naturally derived (such as collagen, chitosan, silk fibroin, alginate, gelatine and hyaluronan)^152^ or synthetic (such as polylactic acid and polyethylene glycol)^152^, have been used to support chondrogenic differentiation. In addition, advanced composite hydrogels offer a more biomimetic approach than traditional injectable hydrogels by incorporating more than one synthetic material and/or key natural components of the ECM^153^; for example, blending collagen hydrogels with GAGs has led to hydrogels that can self-crosslink, which allows for enhanced chondrogenic differentiation^154,155^. In addition, the function of synthetic polymers can be improved with naturally derived ECM components^156^. Further research is needed to understand how additional components, such as other collagen types and small proteoglycans, could be incorporated. These other components have essential roles in fibril formation and stability and could further enhance the biomimicry of injectable hydrogels.

All of these therapeutic strategies for the restoration of articular cartilage incorrectly assume that the collagen network in mature individuals retains the capacity for remodeling^25^. Those developing therapeutic interventions should consider that the collagen network can barely adapt or rebuild in response to injury^145^, and that the collagen synthesis rate by mature chondrocytes is low. With the emergence of rapid prototyping techniques, such as 3D bioprinting, the ability to precisely deposit cell-containing hydrogels (also referred to as bioinks^157,158^) has improved substantially^159^. Moreover, these technologies have enabled the creation of more complex structures that further resemble the native anisotropic tissue composition, potentially enhancing the functionality of engineered articular cartilage-like constructs^35,160,161^. Extrusion-based bioprinting is the most widely used technique^162,163^; however, more advanced printing techniques that can yield higher resolution (such as aerosol jet printing^164^, inkjet bioprinting^165^ and volumetric bioprinting^166^) have also been proposed. Although bioprinting can replicate the depth-dependent chondrocyte density gradients in articular cartilage^164^, it fails to reproduce the collagen type II architecture and proteoglycan organization^167^, leading to tissue with inferior mechanical performance^21^.

To overcome this challenge, hydrogel-based bioprinting has been combined with other biofabrication approaches, such as melt-electrowriting (MEW; Figure 6), to generate articular cartilage implants with improved mechanical performance^168^. MEW utilizes an electric field to 3D print micro-scale polymer fibres, typically using polycaprolactone (PCL), with high resolution. This technique provides flexibility in fibre diameter, deposition patterns and layer stacking^169^, and was successfully combined with multiple biofabrication techniques, including extrusion based bioprinting^170^, inkjet bioprinting^171^ and volumetric printing^172^. The PCL-fibrous network generated by MEW provides a mechanical substitute for the collagen network in the articular cartilage and can remain stable in vivo as demonstrated in horses in which implants remained stable for 6 months^168^. However, this approach is only temporary as PCL is biodegradable and so will slowly disappear and restoration of the collagen network architecture is not induced^25^. Although this issue could potentially be addressed by the inclusion of non-degradable reinforcing polymer fibres (such as polypropylene^173^), true regeneration of the articular cartilage tissue can only be achieved by properly guiding the collagen type II network into its native architecture.

## Attempts to incorporate collagen architecture

The anisotropic organization of the ECM, particularly the alignment of collagen fibres, is essential not only for the mechanical performance of cartilage tissue ^174^ but also for other tissues, such as the intervertebral disc^175^, tendon^126^, cornea^176^, myocardium^177^ and nerves^178^. However, current artificial scaffolds for treating articular cartilage damage cannot replicate the complex collagen type II architecture found in native tissue. Active guidance of collagen type II by chondrocytes has not been yet observed, unlike collagen type I which is guided by fibripositors. Instead, as mentioned previously, the alignment of collagen type II fibres is thought to occur independently of cellular activity^98^. Therefore, if cells cannot directly guide collagen type II deposition, but collagen must be aligned in an arcade-like orientation to provide long-term support to articular cartilage implants, there is a need to address how to align collagen type II. By incorporating collagen fibrillogenesis cues (as discussed earlier in this Review) and orientation methods, it could be possible to improve collagen alignment within 3D-printed articular cartilage constructs, thereby enhancing graft mechanical stability^179^. Incorporating aligned and pre-aligned collagen structures has already shown promising results in promoting cell differentiation^180^ and migration ^181,182^ across various tissues, offering a path toward creating a native-like ECM with long-term functionality and mechanical performance ^25^.

### Approaches to induce collagen alignment

For collagen type I, alignment can be achieved with fluid flow-induced shear forces, which induce collagen monomer orientation along the flow direction^183–187^. Alternatively, tension-based methods^188,189^ (such as dip-pen nanolithography^190^, which relies on fibre stretching), can be used to self-assemble the collagen monomers into networks similar to the natural fibres^191^. Given the challenges of mechanically orienting collagen, alternative approaches have been explored. These approaches include the use of thin aligned fibres obtained with electrospinning^192,193^. Collagen monomers are ejected from a nozzle under the influence of an electric field, leading to the formation of oriented fibres upon solvent volatilization. Wet spinning offers a similar approach, whereby collagen polymer is dissolved in a suitable solvent and extruded through a spinneret into a solvent–non-solvent mixture^194^. Another non-mechanical method for collagen alignment is magnetic alignment^195^. Collagen has diamagnetic properties, which facilitates its perpendicular orientation to the direction of the magnetic field. Similarly, electrochemical alignment involves collagen assembly into fibres perpendicular to the electric field under an electrolysis solution^196^. Other techniques include collagen contact guidance, in which collagen adsorption capacity on the surface of specific substrates is used^181,197^ or molecular crowding, whereby high-concentration collagen fibrillates into specific orientations under confined conditions^198,199^. Collagen strength depends not only on the alignment of the collagen fibres but also on their density, diameter and crosslinking. Research on fibrocartilage has demonstrated that the addition of exogenous LOX results in the alignment of and the formation of collagen fibres that are more mature^200^. Therefore, combining different bioactive stimuli for collagen alignment and fibrillogenesis is a potential strategy to develop functional collagenous tissues for repair and/or replacement in tissue engineering^201^.

The application of these alignment techniques has not yet been explored for collagen type II fibres. In vivo, collagen type II fibres are thinner than collagen type I fibres, with less crystallinity and more binding sites for other collagen types and small proteoglycans ^202–204^. This proteoglycan decoration limits fusion into larger fibres and instead promotes the formation of suprafibrillar assemblies [G] through interconnected bundles. In vitro assembly and fibrillogenesis of collagen type II is governed by liquid crystallinity [G] properties at high concentrations, and the critical concentration for fibre assembly and nucleation rate are considerably different compared with collagen type I^204^. Moreover, although collagen type I fibre growth and orientation are achieved through active cellular processes^97^, self-assembly is cell-independent for collagen type II. Thus, fibre assembly is qualitatively different from collagen type I. Despite these differences most studies investigating the factors that influence collagen alignment in vitro focus on the orientation of collagen type I, and insights into the vitro organization of collagen type II are lacking.

Mechanical loading could serve as a potential stimulus for aligning collagen type II, as it does during postnatal development, in lab-grown articular cartilage scaffolds^205^. For instance, unconfined compression of chondrocyte-seeded agarose scaffolds leads to collagen fibre arrangement that is perpendicular to the direction of loading, therefore leading to fibre alignment parallel to the surface^118^. By contrast, radial confinement also promoted collagen fibre orientation perpendicular to the scaffold surface^206^. Mimicking native compressive forces could offer a mechanical cue for achieving parallel and perpendicular collagen fibre orientation, representing the surface and deep zones of articular cartilage ^205^. This approach was tested by applying mechanical cues to articular cartilage scaffolds using a bioreactor system^209^, which resulted in depth-dependent deposition of ECM components such as GAGs, but did not produce a collagen anisotropic network ^207^. In addition to compression and shear forces, other biomechanical stimuli, such as tension, have been explored. Tensile forces also contribute to articular cartilage homeostasis, and several tensile loading regimens have been reported to induce tissue formation and alignment of collagen fibres with tension^208^. Thus, although mechanically directing collagen type II assembly and orientation remains a challenge, it is one of the most promising approaches. Another area of increasing research is utilizing tissue growth as a potential mechanism for collagen type II alignment; for example, placing tissue-engineered neocartilage (derived from young bovine chondrocyte aggregates) in the upper compartment of a transwell system^101^ and adding TGF-β to the lower compartment (to promote bottom-up ECM deposition) resulted in perpendicular collagen alignment relative to the surface, similar to the alignment observed in the deep articular cartilage zone (Figure 6). However, the observed fibre alignment was limited to certain areas^101^.

### Novel techniques for collagen guidance cartilage

Novel fabrication approaches have helped advance the guidance of collagen type II networks in engineered cartilage implants. Fibrous organized scaffolds generated with MEW technology have been used not only as a direct mechanical reinforcement for hydrogel-based implants^209^ but also as a guiding structure for developing collagenous tissues^210^. Based on this principle, inkjet bioprinting was combined with MEW to precisely deposit cells into MEW-printed PCL grids^171^, which resulted in cell self-assembly into cartilage aggregates that fused together to form hyaline-like tissue^171^ (Figure 6). The collagen network orientation in the final engineered tissue appeared parallel near the surface and curved towards the deep zone. It is probable that the spatial confinement of the fused spherical aggregates within the developing tissue forced the collagen networks along the boundaries of the MEW fibres (Figure 6). Although this approach did give rise to the correct orientation of collagen in some regions, especially the outer regions of the initial aggregates, it still did not reflect the true complexity of the native mature articular cartilage. However, this approach provided important proof that guiding collagen towards its native structure is feasible and could be combined with other additive manufacturing approaches to generate spatially organized osteochondral implants^211^.

Besides MEW, the introduction of the filamented light (FLight) biofabrication technique^212^ further highlights the ability to align collagen type II networks in chondral implants^213^. FLight generates hydrogels with aligned microfilaments and microchannels. These microchannels can promote directional collagen deposition by the cells and results in engineered tissue with aligned collagen and greater mechanical properties (Figure 6). Similarly, aligned rod-shaped microgels have been explored to generate aligned ECM^214^. By using microgels with magnetic properties, the angle of alignment could be controlled and oriented to reproduce the articular cartilage architecture, with the deep zone microgels oriented perpendicular to the surface and superficial zone microgels oriented parallel to the surface^214^ (Figure 6). Other potential approaches include the development of porous scaffolds with anisotropic pore architectures which are designed to direct collagen type II deposition and create biomimetic articular cartilage grafts^215,216^. These novel techniques represent major progress towards the ability to control and guide collagen type II alignment in engineered articular cartilage constructs and could potentially overcome the limitations of current approaches.

## Challenges and considerations

This Review explores the basic biology of articular cartilage, from its cellular and ECM composition in adult tissue to postnatal development that gives rise to the complex tissue architecture. We emphasise the importance of the adult articular cartilage anisotropy and zonation, with a particular focus on the fibrillar collagen network and the proteoglycan-rich matrix. Collagen type II is the most important structural component of the articular matrix, with an organization that gradually changes during postnatal joint morphogenesis leading to a functional arcade-like arrangement^25^. This reorganization of the collagen network, and the proteoglycan matrix, has a key role in the mechanical properties and stability of the native tissue and provides stiffness and compression resilience as the tissue develops, which enables load distribution across the joint and reduces friction during joint movement. Given the poor regenerative capacity of articular cartilage, any damage to the collagen arcade-like structure is potentially irreversible^25^. Therefore, novel approaches, including those using biofabrication, are required to address the mechanical challenges encountered in the restoration of cartilage^217^. Combining these approaches with collagen alignment techniques could offer a promising therapeutic strategy — not only to enhance post-transplant mechanical stability, but also to align collagen in a way that further mimics the native structure and supports long-term function, even after the reinforcement degrades.

Several knowledge gaps remain. Current literature primarily focuses on inducing chondrogenic differentiation for ECM production (both in cell and/or hydrogel-based therapies and in lab-grown implants) and often overlooks the long-term ECM architecture of the resulting tissue. Although this organization is crucial for mechanical properties, the driving factors behind the ECM reorganization during development remain poorly understood. By using polarized light microscopy, many studies describe the transition from an isotropic to an anisotropic collagen network in the adult cartilage^121–123^. Emerging hypotheses suggest that collagen organization during development arises from a combination of appositional tissue growth and mechanical loading that increases postnatally^101^. Applying this knowledge effectively in functional and translational therapies requires a deeper understanding of all the factors that influence this process^25^. For instance, although several mechanotransduction pathways have been identified in chondrocytes, their potential role in postnatal ECM reorganization remains unclear. Additionally, the contribution of other ECM components to this reorganization, such as aggrecan, small proteoglycans and/or remodeling enzymes (such as MMPs and LOX), could pave the way for new therapeutic approaches. Understanding and combining novel technologies with insights into the basic biology of bioactive stimuli that drive ECM organization, such as mechanical loading, bottom-up tissue growth, collagen fibrillogenesis and fibre crosslinking, could lead to the development of tissues with the collagen content, crosslinking level and organization that can recover native mechanical support.

Although this Review primarily focused on structural aspects, it is also important to consider the inflammatory environment that is often present at sites of cartilage defect, especially in OA and traumatic injuries^218^. Inflammatory cytokines and matrix-degrading enzymes can compromise the integrity of both the implanted tissue and any collagen structures within it^219^. Thus, modulating this inflammatory environment through anti-inflammatory treatments or immunomodulatory strategies is essential before transplanting any engineered tissues^218^. Thus, to improve the success and durability of regenerative cartilage restoration, the structural ECM properties of the implant, as well as the inflammatory environment of the host tissue, will need to be addressed.

## Conclusion

As the field of tissue engineering advances, new approaches to chondral implants and collagen guidance will continue to emerge, enabling the development of more biomimetic constructs. By replicating the native collagen architecture and proteoglycan composition of the native articular cartilage, better long-term mechanical stability of implants could be achieved, paving the way for improved post-transplant durability and clinical translation. Insights into the key aspects of articular ECM composition and the structural changes that occur during development can inform tissue engineering therapies. Furthermore, in vitro collagen alignment techniques have the potential to be incorporated into the design of lab-grown implants. Learning from cartilage native architecture could open the possibility of creating mechanically competent and durable tissue constructs that maintain their integrity after transplantation, ultimately helping to reduce the burden of joint disease.

## Supplementary Material

Supplementary Figure 1

## Figures and Tables

**Table 1:** The function of articular cartilage proteoglycans and (glyco)proteins and their interactions with ECM components.

Protein	Function	Interaction with ECM components	Refs
**Proteoglycans**			
Versican	A large bottlebrush chondroitin sulphate-rich proteoglycan. Has a role in mesenchymal condensation and chondrocyte differentiation during development via TGF-β signalling.	Binds to collagen fibres and regulates their structure by increasing collagen condensation. Interacts with hyaluronan, fibrillin, tenascin, fibronectin and heparan sulphate rich-proteoglycans to aid ECM crosslinking.	^220,221^
Perlecan	A PCM heparan sulphate-rich proteoglycan. Binds growth factors, such as FGF, to promote chondrogenesis. Contributes to ECM mechanical stability.	Interacts with collagen type VI and collagen type XI and other cell adhesive and structural glycoproteins to stabilize the chondrocyte PCM.	^222–224^
Lubricin (PRG-4)	A mucinous glycoprotein on the articular cartilage surface. Provides lubrication to the joint. Protects articular cartilage from cell adhesion, inhibits synovial overgrowth and prevents cartilage–cartilage adhesion.	Interacts with COMP, collagen type II and fibronectin in the superficial zone of the articular cartilage.	^225–228^
Fibromodulin^a^	A keratan sulphate-rich proteoglycan. Has an important role during development and mesenchymal condensation. Surrounds cells at the articular cartilage surface during development and is becoming more widely detected across postnatal ECM.	In adult articular cartilage, its levels correlate with the size of collagen fibres. Influences fibrillogenesis and crosslinking by connecting collagen fibres with LOX.	^229,230^
Lumican^a^	A keratan sulphate-rich proteoglycan. Participates in host–pathogen interactions.	Interacts with and restricts the growth of collagen type II fibres.	^231,232^
Decorin^a^	A small proteoglycan with one GAG chain of chondroitin sulphate or dermatan sulphate. Forms rings along the collagen fibres. Its absence leads to a disorganized collagen network.	Binds non-covalently to collagen fibres hindering the binding of other collagen molecules. Regulates the lateral growth of collagen fibres, which controls fibre diameter.	^233^
Biglycan^a^	A small proteoglycan with two GAG chains either chondroitin sulphate or dermatan sulphate. Has a role in collagen type II fibre formation. Regulates morphogenesis and differentiation in the PMC.	Regulates collagen fibre diameter. Together with decorin, interacts with matrilin-1 to link collagen type VI (which organizes into beaded filaments that form hexagonal networks) to aggrecan and collagen type II.	^84,233^
Chondroadherin^a^	A small cartilage matrix protein that mediates chondrocyte adhesion. Has a role in cartilage homeostasis and morphogenesis. Localized in the territorial matrix during development.	Regulates cell–matrix interactions by binding collagen type II and collagen type VI to cell receptors (such as integrins and cell surface proteoglycans).	^234,235^
PRELP^a^	A proline and arginine-rich end leucine-rich proteoglycan. Acts as a link between the ECM and cells in articular cartilage.	Binds collagen and perlecan, acting as a link between the PCM and the territorial matrix.	^236^
**Other (glyco)proteins**			
Matrilins	An adaptor protein for collagen and proteoglycans. Pericellular matrilins allow chondrocyte mechanotransduction. Has role in the maintenance of chondrogenesis.	In native articular cartilage these proteins bind mainly to collagen type IX on the fibre surface. Both matrilin-1 and matrilin-3 regulate lateral collagen fibre growth.	^237^
Thrombo spondin	A matricellular protein. Mediates cell–cell and cell–ECM interactions. Has a role in articular cartilage formation and chondrogenesis.	Regulates collagen fibre assembly by regulating collagen crosslinking enzymes, such as LOX.	^238^
COMP	A pentameric glycoprotein, also known as thrombospondin-5. Involved in collagen organization. Binds to ECM components, such as collagen, aggrecan, matrilins and fibronectin. Involved in early limb development by binding to TGF**-**β and BMPs.	Interacts and regulates collagen type II fibrillogenesis. Enhances fibre formation by bringing collagen together and catalysing fibrillogenesis with a uniformed diameter.	^239,240^
Fibrillin-1	A large ECM glycoprotein that forms a fibre meshwork within cartilage. Together with elastin, contributes to the poroelasticity of the articular cartilage. Its microfibrils regulate TGF-β bioavailability in the PCM (which in turn inhibits chondrocyte hypertrophy).	Involved in cell-ECM adhesion. Interacts with perlecan and other molecules that attach to collagen.	^241–243^
Elastin	A protein found in the ECM. Provides tissue flexibility and maintains the elasticity of articular cartilage. Can be involved in cell adhesion, migration and signalling.	Bridging molecules anchor elastin fibres to collagen. At the collagen surface, elastin fibres are oriented parallel to the collagen type II fibres; elastin is not detected in the deep zone.	^244,245^
Tenascin-C	A glycoprotein involved in chondrogenesis during embryonic development. Remains as an ECM component postnatally.	Interacts with collagen type IX, acting as a link between chondrocytes and the ECM. Connects chondrocytes and the surrounding ECM by binding to cell receptors.	^246^
Fibronectin	A multidomain glycoprotein that forms a fibrillar network and requires the support of cell integrins for assembly. Regulates ECM remodeling through interaction with α5β1 integrins.	Binds to collagen type II and heparan sulphate proteoglycans, providing binding sites for cell adhesion proteins (such as syndecans and integrins).	^247,248^
Laminins	A large glycoprotein secreted by chondrocytes and located in the PCM. Involved in chondrocyte adhesion, migration and resistance to apoptosis.	Promotes chondrogenesis by increasing collagen type II, COMP and aggrecan production.	^249,250^

a indicates small leucine-rich proteoglycans

BMP, bone morphogenetic protein; ECM, extracellular matrix; FGF, fibroblast growth factor; GAG, glycosaminoglycan; LOX, lysyl oxidase; PCM, pericellular matrix; PRELP, proline/arginine-rich end leucine-rich proteoglycan; TGF-β, transforming growth factor-beta; COMP, cartilage oligomeric matrix protein.

## Key points

- Articular cartilage is a highly anisotropic tissue that is characterized by depth-dependent collagen fibre orientation which provides the tissue with its unique biomechanical properties.
- The collagen architecture of the articular cartilage forms during late foetal and postnatal development in response to biomechanical stimuli and tissue growth.
- Collagen interacts with the surrounding extracellular matrix (ECM), including aggrecan, by limiting the water swelling pressure caused by negatively charged glycosaminoglycans, thereby enhancing cartilage resilience.
- Small proteoglycans and (glyco)proteins promote tissue cohesion and stability by regulating fibrillogenesis and connecting various ECM components together.
- Achieving native-like collagen anisotropy in engineered cartilage remains a challenge, as current scaffolds cannot replicate the depth-dependent alignment of collagen and its ECM interactions.
- Insights gained from cartilage development and its anisotropic organization could inspire novel strategies for achieving long-term cartilage regeneration.

## Glossary

### Abbreviations

Heterotypic fibres: Collagen fibres composed of more than one type of collagen molecule.
Arcade-like structure: A collagen fibre arrangement in articular cartilage whereby fibres curve from the surface to the deep zone, anchoring to the subchondral bone.
Anisotropic: Having direction-dependent properties.
Isotropic: Having uniform properties in all directions.
Split-line orientation pattern: A pattern observed on the surface of cartilage that reflects the underlying collagen fibre alignment.
Beaded microfibrillar network: A structural arrangement of thin, bead-like fibrils connected in a network.
Joint cavitation: The formation of the joint cavity during embryonic development, separating the joint into two articular surfaces.
Nascent: Newly formed or immature.
Tidemark: Boundary between the calcified and non-calcified zones of articular cartilage.
Hypertrophy: The process by which cells undergo significant enlargement due to volumetric increase and distinct metabolic and molecular changes.
Fibrillogenesis: The process of collagen fibril formation.
Procollagen: The precursor molecule of collagen that undergoes enzymatic processing to form tropocollagen.
Tropocollagen: The basic triple-helical collagen molecule that assembles into fibrils.
Nucleator: A molecule or structure that promotes the initiation of fibrillogenesis.
Reducible crosslinks: A reversible chemical bond that stabilizes collagen fibrils.
Non-reducible crosslinks: A non-reversible chemical bond that permanently stabilizes collagen fibrils.
Resistance to the positive principal strain: The ability of a material to withstand tensile forces along the direction of the highest principal strain.
Molecular crowding: A high concentration of macromolecules in a confined space that influences molecular interactions and assembly.
Suprafibrillar assemblies: Higher-order structures formed by the organization of fibrils into complex networks.
Liquid crystallinity: A state of matter with properties between those of a liquid and a solid crystal, characterized by ordered molecular alignment.
